# Abatacept as maintenance therapy in kidney transplantation: a retrospective comparative study

**DOI:** 10.3389/ti.2026.16366

**Published:** 2026-09-14

**Authors:** Charlotte Uro-Coste, Inès Ramos, Aurélien Tiple, Marine Freist, Claire Chatron, Alba Atenza, Jimmy Garraud, Clarisse Grèze, Carole Philipponnet, Paul Rouzaire, Anne-elisabeth Heng, Cyril Garrouste

**Affiliations:** 1 Université Clermont Auvergne, Clermont-Ferrand, France; 2 Département de Néphrologie, Centre Hospitalier Universitaire de Clermont-Ferrand, Clermont-Ferrand, France; 3 Département de Biostatistiques, Centre Hospitalier Universitaire de Clermont-Ferrand, Clermont-Ferrand, France; 4 Département de néphrologie, centre hospitalier de Vichy, Vichy, France; 5 Département de Néphrologie, Centre Hospitalier du Puy-en-Velay, Le puyenvelay, France; 6 Pharmacie Hospitaliére, Centre Hospitlier Universitaire de Clermont-Ferrand, Clermont-Ferrand, France; 7 Département d'immunologie, Centre Hospitalier Universitaire de Clermont-Ferrand, Clermont-Ferrand, France

**Keywords:** abatacept, belatacept, calcineurin inhibitor toxicity, immunosuppressive therapy, kidney transplantation

## Abstract

Belatacept use is increasinging in kidney transplantation, as it has demonstrated improved graft survival compared with calcineurin inhibitors. However, its intravenous administration and recurrent supply shortages may limit its use. Abatacept has a similar mechanism of action. Although widely used in rheumatology, its use in kidney transplantation remains poorly documented. This study aimed to compare the efficacy and safety of abatacept and belatacept as maintenance immunosuppressive therapy after kidney transplantation. We conducted a retrospective study including adult kidney transplant recipients who were switched to abatacept or belatacept between 2017 and 2023. The primary endpoint was the occurrence of rejection within the first year after conversion. A total of 74 patients were included: 21 in the abatacept group and 53 in the belatacept group. No significant difference was observed in rejection rates at 1 year (respectivily 9.5% vs. 9.4%; p > 0.999). Rates of viral and opportunistic infections were also comparable. These findings, which warrant confirmation in larger studies, suggest that abatacept is associated with acceptable safety outcomes relative to belatacept. Abatacept may thererefore be considered a potential alternative in selected situations, including drug shortage, limited venous access, pregnancy or the need for greater treatment autonomy.

## Graphical Abstract

**Figure:**
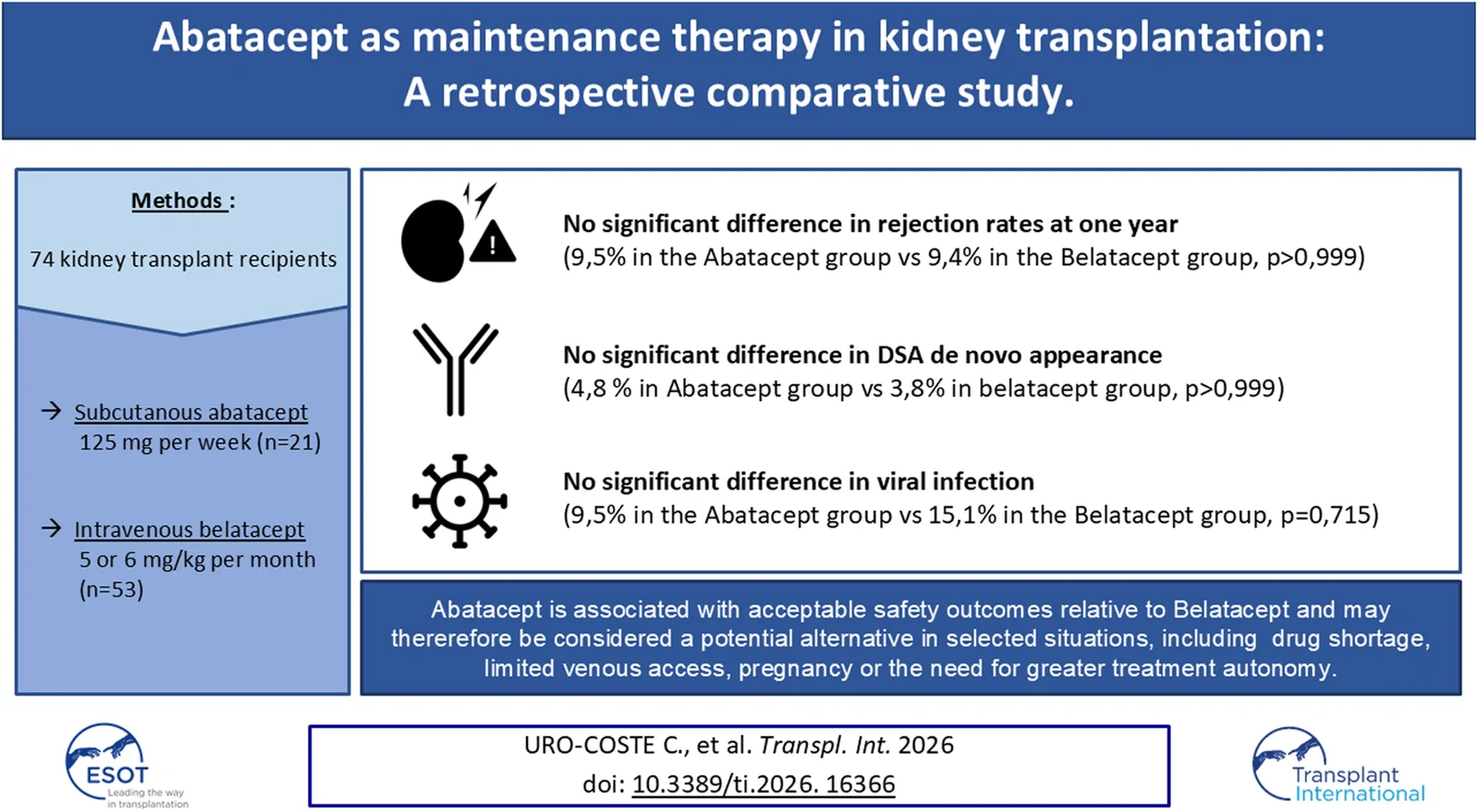

## Introduction

Abatacept is an immunosuppressive treatment widely used in rheumatology, especially in the management of rheumatoid arthritis [1]. This fusion protein is composed of the constant region of the human IgG1 heavy chain, linked to the extracellular domain of human cytotoxic T-lymphocyte-associated antigen 4 (CTLA-4). This structure allows selective inhibition of T-cell activation by blocking the CD28-CD80/86 costimulatory pathway. Abatacept can be administered either subcutaneously or intravenously.

Belatacept has a mechanism of action similar to that of Abatacept. Its composition differs by only 2 amino acids, making it more affine to its ligand. It was developed as an alternative to calcineurin inhibitors (CNIs) in kidney transplantation, as CNI-associated vascular toxicity contributes to accelerated kidney graft ageing [2]. Following its approval by the U.S. Food and Drug Administration and the European Medicines Agency in 2011, belatacept use has become increasingly widespread, as it has demonstrated superior outcomes in renal transplant (RT) survival and improved long-term renal function, both in induction therapy and as an alternative to CNI treatment [3–5]. More recently, some studies have highlighted the potential benefit of using belatacept, in some cases in combination with low-dose CNIs, as maintenance therapy for patients with immunological challenges, such as those related to the presence of donor-specific antibodies (DSAs) or chronic antibody-mediated rejection (cAMR) [6–11].

However, there are certain considerations regarding the use of belatacept. It is administered exclusively intravenously, which can be challenging for patients with chronic kidney disease who often have limited peripheral venous access. Moreover, belatacept has at times become unavailable because of production shortages. There is therefore considerable interest in finding a therapeutic alternative.

Despite significant similarities between belatacept and abatacept, the latter has rarely been studied in the context of renal transplantation. To date, there are 3 case series concerning its use in this context [12–14], whose findings are generally encouraging in terms of safety and efficacy.

This study aimed to assess the safety and efficacy of abatacept in comparison with belatacept over the course of the first year following treatment conversion.

## Materials and methods

### Population and general data

We conducted a retrospective observational study in 3 centres in the Auvergne Region (Hôpital Universitaire de Clermont-Ferrand, Hôpital de Vichy, Hôpital du Puy-en-Velay). We included all kidney transplant patients over 18 years old who were switched to abatacept or belatacept between January 2017 and June 2023. Patient characteristics and biological data were collected from electronic medical records.

This study was approved by the local Ethics Committee (IRB00013412, “CHU de Clermont Ferrand IRB #1”, IRB 2024-CF273) and complied with the French policy of individual data protection.

### Definition of groups

Patients in the belatacept group received intravenous (IV) belatacept (5 or 6 mg/kg according to the pharmaceutical company’s recommendations at the time of treatment); they received one injection every 2 weeks for 2 months during the transition phase, followed by monthly maintenance infusions. The initial infusions were administered in a hospital setting, while the maintenance infusions were given either in the hospital or at the patients' homes.

Patients in the abatacept group received subcutaneous abatacept (125 mg per week). The first injection was administered in a hospital setting, after which the treatment was continued at home. Two patients had been receiving belatacept therapy prior to conversion to abatacept—one for 18 months and the other for 7 years. The first dose of abatacept was administered 1 month after the last infusion of belatacept.

### End points and event definition

The primary endpoint was the occurrence of rejection within the first year post-conversion. Rejection was defined as either T-cell–mediated rejection (TCMR) or antibody-mediated rejection according to the Banff criteria. No protocol biopsies were performed following conversion to CTLA4-Ig; indication biopsies were carried out at the clinician’s discretion, typically in cases of graft dysfunction, new-onset proteinuria, or the appearance or an increased level of DSAs.

Secondary endpoints included DSA appearance and an increase in DSA levels. Anti-HLA antibody levels were measured using a LUMINEX-based assay at 3, 6, and 12 months after conversion to belatacept or abatacept or in cases of graft dysfunction. A mean fluorescence intensity (MFI) threshold of 1000 was used to define DSA positivity; a significant increase in the DSA level was defined as a >50% increase in the MFI compared with the preconversion value.

### Statistical analysis

Statistical analyses were performed using R software (version 4.3.1; R Core Team, 2023; R Foundation for Statistical Computing, Vienna, Austria), considering a two-sided type I error rate of 5%. Categorical variables are presented as counts and associated proportions, and quantitative variables are presented as the mean ± standard deviation or median [Q1, Q3], depending on their distribution. Data between groups (abatacept vs. belatacept) were compared using Student’s t*-*test or the Mann–Whitney test for nonnormally distributed quantitative variables and the chi-square test or Fisher’s exact test for categorical variables, as appropriate.

## Results

### Patient characteristics

A total of 74 patients were included in our study: 21 were in the abatacept group, and 53 were in the belatacept group. Patient characteristics, transplantation details, and parameters related to conversion to costimulation inhibitors are given in Table 1. Our cohort consisted of 53 men (71.6%) with a mean age of 49.1 years. The majority of patients received induction therapy with basiliximab: 13 patients (61.9%) in the abatacept group and 38 patients (71.7%) in the belatacept group (p = 0.412). Twelve patients (16.2%) had a preformed DSA at the time of transplantation.

**TABLE 1:** Patient characteristics, transplantation details, and parameters related to conversion to costimulation inhibitors.

Characteristics	Overall N = 74	Abatacept N = 21	Belatacept N = 53	p value
Age at transplantation	49.1 ± 18.2	50.0 ± 20.7	48.8 ± 17.2	0.640
Male sex	53 (71.6%)	15 (71.4%)	38 (71.7%)	0.982
Causal nephropathy				
Glomerulonephritis	22 (29.7%)	5 (23.8%)	17 (32.1%)	0.483
Genetic cause	19 (25.7%)	7 (33.3%)	12 (22.6%)	0.384
Diabetes	8 (10.8%)	3 (14.3%)	5 (9.4%)	0.680
Vascular nephropathy	7 (9.5%)	2 (9.5%)	5 (9.4%)	>0.999
Interstitial nephritis	8 (10.8%)	1 (4.8%)	7 (13.2%)	0.427
Unknown	7 (9.5%)	2 (9.5%)	5 (9.4%)	>0.999
Others	3 (4.1%)	1 (4.8%)	2 (3.8%)	>0.999
Retransplantation	9 (12.2%)	2 (9.5%)	7 (13.2%)	>0.999
Induction therapy	​	​	​	0.412
Basiliximab	51 (68.9%)	13 (61.9%)	38 (71.7%)	​
Antithymocyte globulin	23 (31.1%)	8 (38.1%)	15 (28.3%)	​
CMV status				
CMV donor -/recipient -	12 (16.2%)	5 (23.8%)	7 (13.2%)	0.303
CMV donor +/recipient -	21 (28.4%)	8 (38.1%)	13 (24.5%)	0.243
CMV recipient +	39 (52.7%)	8 (38.1%)	31 (58.5%)	0.113
DSA at transplantation	12 (16.2%)	4 (19.0%)	8 (15.1%)	0.731
cPRA	​	​	​	0.817
<5	57 (77.0%)	16 (76.2%)	41 (77.4%)	​
5–85	8 (10.8%)	3 (14.3%)	5 (9.4%)	​
>85	9 (12.2%)	2 (9.5%)	7 (13.2%)	​
Time of conversion after transplantation	34.1 [10.6, 83.2]	44.0 [13.0, 92.5]	32.6 [9.3, 78.7]	0.465
Conversion indication				
CNI toxicity/marginal graft	51 (68.9%)	14 (66.7%)	37 (69.8%)	0.891
CAMR	17 (23.0%)	6 (28.6%)	11 (20.8%)	0.763
*De novo* DSA	2 (2.7%)	0 (0.0%)	2 (3.8%)	>0.999
Gastroparesis	2 (2.7%)	1 (4.8%)	1 (1.9%)	0.490
Medication nonadherence	1 (1.4%)	0 (0.0%)	1 (1.9%)	>0.999
Multiple medication intolerance	1 (1.4%)	0 (0.0%)	1 (1.9%)	>0.999
Reason for choosing abatacept				
Belatacept unavailability	—	13 (61.9%)	—	—
Difficult venous access	—	6 (28.6%)	—	—
COVID-19 pandemic	—	2 (9.5%)	—	—
Associated treatment				
Tacrolimus	15 (20.3%)	7 (33.3%)	8 (15.1%)	0.109
MMF	59 (79.7%)	15 (71.4%)	44 (83.0%)	0.338
mTORi	7 (9.5%)	3 (14.3%)	4 (7.5%)	0.397
Corticosteroids	64 (86.5%)	14 (66.7%)	50 (94.3%)	0.004
Azathioprin	4 (5.4%)	2 (9.5%)	2 (3.8%)	0.318

Data are presented as the numbers (percentages) of patients, means ± standard deviations or medians [25th; 75th percentiles]. CAMR: chronic antibody-mediated rejection, CMV: cytomegalovirus, CNI: calcineurin inhibitor, cPRA: calculated panel reactive antibody, DSA: donor-specific antibody, MMF: mycophenolate mofetil, mTORi: mammalian target of rapamycin inhibitor.

Conversion to CTLA4-Ig occurred at a mean of 34.1 months (range: 10.6–83.2) post-transplantation—44 months (13–92.5) in the abatacept group and 32.6 months (9.3–78.7) in the belatacept group (p = 0.465). Conversion was prompted by CNI toxicity or a marginal graft (68.9% of cases), cAMR (23% of cases), the emergence of a DSA (2.7% of cases), gastroparesis (2.7% of cases), nonadherence to oral immunosuppressive therapy (1.4% of cases), or intolerance to multiple immunosuppressive agents (1.4% of cases). The reasons for conversion did not differ significantly between the two groups.

Abatacept was selected over belatacept mainly because of a shortage of belatacept (n = 13, 61.9%). In 28.6% of the cases (n = 6), the choice was driven by issues related to vascular access, whereas in 2 patients (9.5%), abatacept was preferred to minimize hospital visits during the COVID-19 pandemic.

Regarding the immunosuppressive regimen administered in combination with CTLA4-Ig, 15 patients (20.3%) were maintained on tacrolimus, aiming for a target trough concentration between 2 and 5 ng/mL. A greater proportion of patients in the abatacept group were on maintenance tacrolimus, although this difference was not statistically significant (33.3% in the abatacept group vs. 15.1% in the belatacept group; p = 0.109). Corticosteroids were more frequently prescribed in the belatacept group (94.3%) than in the abatacept group (66.7%) (p = 0.004). Overall, 59 patients (79.7%) received mycophenolate mofetil (MMF), and 7 patients (9.5%) were treated with a mammalian target of rapamycin inhibitor (mTORi). The distributions of these two agents were comparable between the two groups.

### Rejection and DSA

The rejection rates within the first year following conversion to abatacept or belatacept were similar between the two groups: 2 episodes (9.5%) in the abatacept group and 5 episodes (9.4%) in the belatacept group (p > 0.999) (Table 2). The characteristics of patients who experienced rejection, as well as details of the rejection episodes, are presented in Table 3. Only one patient, in the belatacept group, experienced rejection with microvascular inflammation in the absence of DSA; all other episodes were TCMR.

**TABLE 2:** Occurrence of rejection within the first year post-conversion and secondary endpoints.

Endpoints	Total n = 74	Abatacept n = 21	Belatacept n = 53	p value
Rejection	7 (9.5%)	2 (9.5%)	5 (9.4%)	>0.999
TCMR	6	2	4	​
ABMR	1	0	1	​
Secondary endpoints				
DSA *de novo*	3 (4.1%)	1 (4.8%)	2 (3.8%)	>0.999
Increased in DSA levels	2 (2.7%)	2 (9.5%)	0 (0.0%)	0.078
Viral complication	10 (13.5%)	2 (9.5%)	8 (15.1%)	0.715
- BK virus replication	1 (1.4%)	0 (0.0%)	1 (12.5%)	​
- Severe COVID-19	1 (1.4%)	0 (0.0%)	1 (12.5%)	​
- CMV disease	6 (60.0%)	1 (50.0%)	5 (62.5%)	​
- Norovirus infection	1 (1.4%)	0 (0.0%)	1 (12.5%)	​
- Herpes zoster	1 (1.4%)	1 (50.0%)	0 (0.0%)	​

Data are presented as the numbers (percentages) of patients. CMV: cytomegalovirus, DSA: donor-specific antibody.

**TABLE 3:** Patients who experienced rejection: characteristics and details.

​	Abatacept		Belatacept				
Characteristics	Patient 1	Patient 2	Patient 3	Patient 4	Patient 5	Patient 6	Patient 7
Age at Tx (years)	72	48	72	46	71	44	68
Sex	F	M	F	M	F	F	M
Induction therapy	Basiliximab	ATG	Basiliximab	Basiliximab	ATG	Basiliximab	Basiliximab
Conversion indication	Marginal graft	CNI toxicity	Marginal graft	CNI toxicity	CNI toxicity	CNI toxicity	CNI toxicity
Time of conversion after Tx	1 month	16 months	Induction treatment	6 months	11 months	15 months	3 months
Delay CNI discontinuation after conversion	2 weeks	1 month	No CNI	1 month	3 weeks	1 month	3 weeks
Cause of graft biopsy	3 months protocol biopsy	Graft dysfunction	3 months protocol biopsy	Graft dysfunction	Graft dysfunction	Graft dysfunction	Graft dysfunction
Rejection type	Cellular 1B	Cellular 1A	Cellular 1B	Cellular 2B	MVI without DSA Suspicious AMR	Borderline	Cellular 1A
Time from conversion to rejection	2 months	8 months	3 months	3 months	2 months	3 months	3 months
Associated treatment at time of rejection	MMF 750 mg/d Prednisone 7.5 mg/d	mTORi Prednisone 5 mg/d	MMF 1500 mg/d Prednisone 5 mg/d	MMF 2 g/d, prednisone 5 mg/d	MMF 750 mg/d	AZA 100 mg/d, prednisone 5 mg/d	MMF 2 g/d, prednisone 5 mg/d
Treatment	Pulse steroids followed by oral steroids at 1 mg/kg/d tapered over 3 months	- Pulse steroids followed by oral steroids at 1 mg/kg/d tapered over 3 months - Switch mTORi to low dose CNI	Pulse steroids followed by oral steroids at 1 mg/kg/d tapered over 3 months	- ATG - Pulse steroids followed by oral steroids at 1 mg/kg/d tapered over 3 months - Low dose CNI	- Plasmapheresis - Pulse steroids followed by oral steroids at 1 mg/kg/d tapered over 3 months - Low dose CNI	- Pulse steroids followed by oral steroids at 1 mg/kg/d tapered over 3 months - Switch AZA to MMF	- Pulse steroids followed by oral steroids at 1 mg/kg/d tapered over 3 months
Re-evaluation graft biopsy	Resolution of graft inflammation at 3 months	Resolution of graft inflammation at 3 months	No re-evaluation graft biopsy	No re-evaluation graft biopsy	Advanced renal fibrosis	No re-evaluation graft biopsy	No re-evaluation graft biopsy
Evolution of graft function	SCr 70 μmol/L at biopsy; stable at 3 and 12 months	SCr 300 μmol/L at biopsy (baseline 200) 250 at 3 months and 1 year	SCr 80 μmol/L at biopsy; stable at 3 and 12 months	SCr 380 μmol/L, 200 at 3 months and 1 year	SCr 800 μmol/L at biopsy, dialysis requirement at 4 months	SCr 350 μmol/L at biopsy, 200 at 3 months	SCr 250 μmol/L at biopsy, 140 at 2 weeks

ATG: antithymocyte globulin, AZA: azathioprine, SCr: serum creatinine, CNI: calcineurin inhibitor, DSA: donor-specific antibody F: female, M: male, MMF: mycophenolate mofetil, mTORi: mammalian target of rapamycin inhibitor, MVI: microvascular inflammation, Tx: transplantation.

DSAs appeared in 3 patients within the year following conversion: one patient in the abatacept group developed, 12 months after conversion, an anti-B8 DSA with a MFI of 1700, without histological signs of rejection on graft biopsy, and 2 patients in the belatacept group developed DSAs—one with anti-Cw2 (MFI 1200) and the other with anti-A11 (MFI 1000) at 3 months and 12 months, respectively, after conversion.

A significant increase in DSA was observed in two patients in the abatacept group. In both patients, the increase involved an anti-DQ antibody. The MFI rose from 1,400 to 2,100 in the first patient and from 2,000 to 8,000 in the second. The second patient was also the one in whom the emergence of an anti-B8 antibody was observed.

### Viral complications and opportunistic infection

Ten patients (13.5%) experienced viral complications: 2 patients (9.5%) in the abatacept group and 8 patients (15.1%) in the belatacept group (p > 0.999). Details of the infections are presented in Table 2.

In the abatacept group, one patient experienced CMV colitis with a viral load of 6 log copies/mL 5 months after the initiation of abatacept in a 76-year-old patient with positive CMV serology, in whom CMV prophylaxis had been discontinued. The second infection was a case of zoster reactivation occurring 1 month after starting abatacept in a 57-year-old patient, who achieved a favourable outcome under valaciclovir treatment.

Within the cohort, only one case of nonviral opportunistic infection was reported, occurring in a patient from the belatacept group who presented with cryptosporidium-induced diarrhoea 4 months after conversion. All patients received cotrimoxazole after CTLA4-Ig conversion.

### Changes in the estimated glomerular filtration rate (eGFR) at 12 months

A global increase in the eGFR was observed in both groups following the introduction of abatacept or belatacept. In the abatacept group, the increase was 2.3 (±11.6) mL/min/1.73 m^2^ at 3 months and 2.9 (±12.9) mL/min/1.73 m^2^ at 1 year, whereas in the belatacept group, it was 3.8 (±10.3) mL/min/1.73 m^2^ at 3 months and 4.5 (±15.2) mL/min/1.73 m^2^ at 1 year. There was no statistically significant difference in eGFR evolution between the two groups. No patient experienced graft loss.

## Discussion

To our knowledge, this is the first study directly comparing abatacept and belatacept as maintenance immunosuppressive therapy following kidney transplantation. Our findings provide reassuring evidence indicating the absence of an increased risk of rejection following conversion to abatacept. The observed acute rejection rate of 9.5% within the first year post-conversion was comparable to that observed following belatacept conversion in our cohort, as well as to rates previously reported in the literature. For instance, in a prospective study, Budde et al. reported an 8% incidence of acute cellular rejection at 24 months following late conversion to belatacept (>6 months post-transplant) [4]. Similarly, two European multicentre retrospective studies, separately authored by Bertrand et al. and Darres et al., reported rejection rates of 6.1%—predominantly occurring within the first 3 months post-conversion—and 8.2%, respectively [4, 5, 15]. Across all these studies, most rejection episodes occurred within the first year after conversion, supporting our decision to use this timeframe as the primary endpoint. In our cohort, both rejection episodes following conversion to abatacept were TCMR. One of these patients had been converted early, at 3 months post-transplant, as suggested in the literature to potentially improve graft survival—although this timing is also considered a risk factor for rejection [15, 16]. In both cases, follow-up biopsies performed 3 months after rejection and subsequent treatment showed complete resolution of inflammation, allowing continuation of abatacept therapy [15]. In our cohort, tacrolimus was maintained in 7 of 21 patients (33.3%) receiving abatacept, with low target trough levels (2–4 ng/mL) [17]. This combination may have contributed to the low rejection rate. In 5 of these 7 patients, the decision to maintain tacrolimus was motivated by a history of cAMR prior to the initiation of abatacept [6–11]. In addition, it should be noted that, as in most studies and case series addressing conversion to a costimulation inhibitor [3–5, 7, 18], no systematic biopsies were performed in either the abatacept or belatacept groups, raising the possibility of undiagnosed subclinical rejection. However, the observed stability of renal function at 1-year-follow-up in patients who did not undergo biopsy may be considered a reassuring finding.

To date, only three case series have documented the use of abatacept in kidney transplantation. Badell et al. described the use of IV abatacept (10 mg/kg/month) in 9 recipients with a median follow-up of 82 months [14]. Only one patient experienced favourable-outcome cellular rejection when treated with corticosteroids, while renal function and panel reactive antibody (PRA) levels remained stable in all patients. We previously reported the first case series of five patients who converted to subcutaneous abatacept, none of whom experienced rejection [12]. The largest series to date, published by Bertrand et al., involved 171 patients who received abatacept for 3 months during the COVID-19 pandemic to avoid in-hospital belatacept infusions, which were not permitted in outpatient settings at the time [13]. The authors reported reassuring results, with stable graft function in 97.2% of the recipients. It is noteworthy that, in the three aforementioned series, the indication for conversion to CTLA-4 inhibitors was exclusively [14] or predominantly [12, 13] intolerance to calcineurin inhibitors, encompassing both CNI-related toxicity and the use of marginal grafts. In contrast, indications for conversion in our study were more heterogeneous, resulting in a more diverse cohort. Nevertheless, this heterogeneity may strengthen the external validity of our findings, as it more accurately reflects the contemporary, diverse use of CTLA-4 inhibitors in clinical practice [6–11].

While the risk of opportunistic infections following conversion to belatacept has been frequently reported in the literature [19, 20], few infectious events were observed in our cohort, whether in the abatacept or belatacept groups. In terms of its primary indication, rheumatoid arthritis, the reported infectious risk of abatacept remains modest [21]; however, extrapolation to transplant recipients must be made with caution, given the concomitant use of immunosuppressive therapy. A lower infection risk with abatacept than with belatacept could be hypothesized, given its lower binding affinity for CD80/CD86, but this requires confirmation in larger studies with extended follow-up.

Several practical considerations motivated the use of abatacept in our centre. Following the COVID-19 pandemic and in the context of promoting outpatient care for patients with significant CNI-induced nephrotoxicity, the most frequent reason for abatacept use in our cohort was the national shortage of belatacept in France from January to September 2023. An especially valuable indication may be its suitability for patients with poor venous access, for whom subcutaneous administration offers clear advantages. While belatacept was administered intravenously by a nurse for all patients in that treatment group, abatacept was self-administered via subcutaneous injections by all patients in that group, offering the patients greater autonomy. This benefit was also highlighted in a study by Bertrand et al., in which 47% of 177 patients who converted to abatacept during the COVID-19 pandemic reported that treatment was less burdensome than belatacept was, and 38% expressed a preference to continue abatacept therapy [13]. Finally, although recent data have provided reassuring evidence regarding the use of belatacept during pregnancy [22], more extensive and longer-term safety data are currently available for abatacept in this setting [23, 24].

In conclusion, this real-world study suggests that abatacept is associated with acceptable safety outcomes compared with belatacept, without evidence of an increased risk of rejection. These findings support its consideration as a potential alternative, particularly in the context of drug shortages, for patients with difficult venous access, during pregnancy, or for those seeking greater treatment autonomy. Additional data on the safety and efficacy of this strategy are expected from an ongoing randomized prospective trial [25].

## Data Availability

The raw data supporting the conclusions of this article will be made available by the authors, without undue reservation.
